# Supplementary material for: Taxonomic analysis of metagenomic data with kASA
Source: Nucleic Acids Res. 2021 Mar 30;49(12):e68. doi: 10.1093/nar/gkab200 (PMC8266618; doi:10.1093/nar/gkab200)
Supplement: gkab200_Supplemental_Files [file gkab200_supplemental_files.zip › Suppl_D.zip!/OPAL_comparison_new/plot_shannon.pdf]

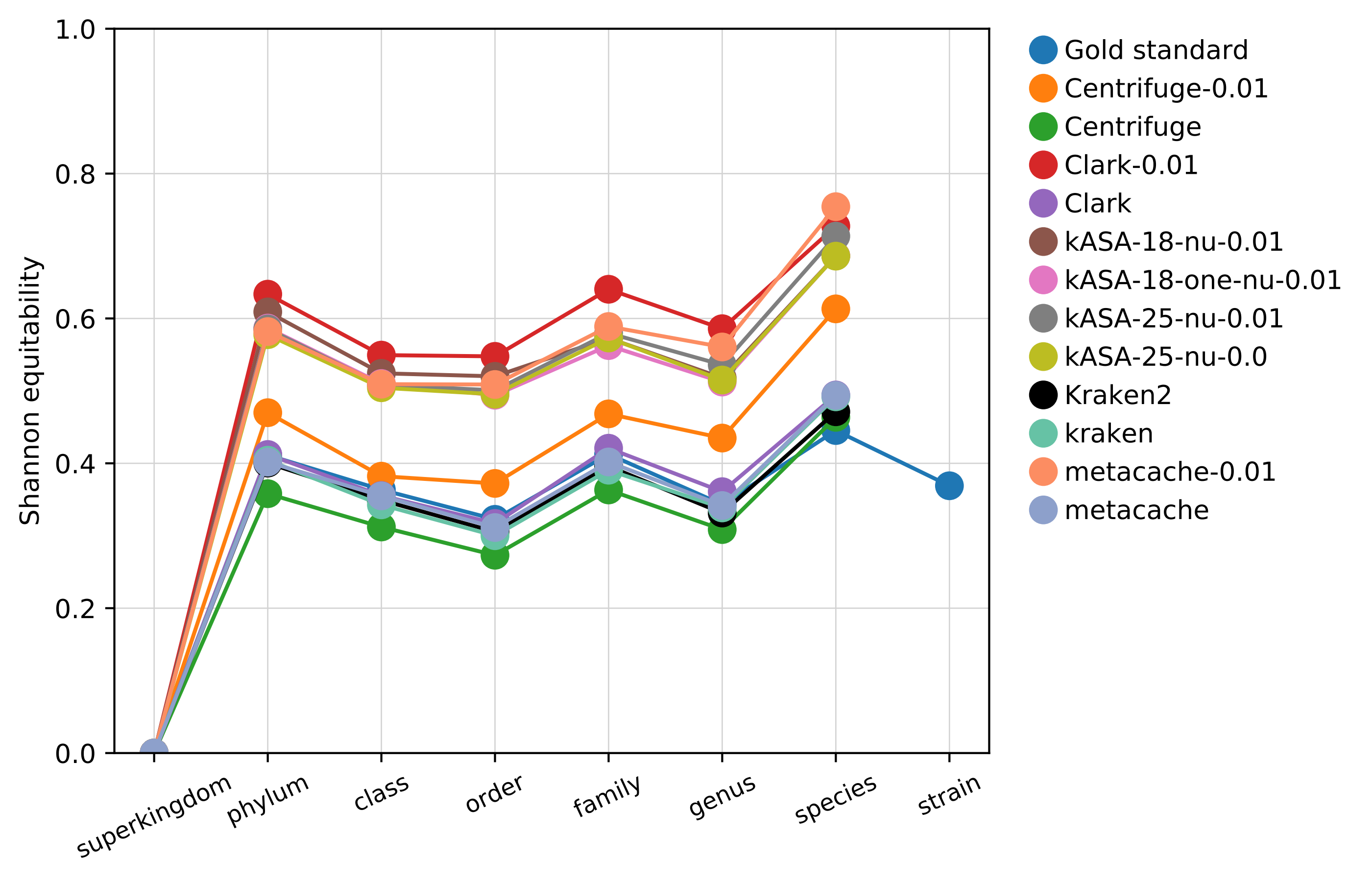

Shannon equitability
0.0
0.2
0.4
0.6
0.8
1.0
superkingdom
phylum
class
order
family
genus
species
strain
Gold standard
Centrifuge-0.01
Centrifuge
Clark-0.01
Clark
kASA-18-nu-0.01
kASA-18-one-nu-0.01
kASA-25-nu-0.01
kASA-25-nu-0.0
Kraken2
kraken
metacache-0.01
metacache
